# Three-Dimensional Heads-Up vs. Standard Operating Microscope for Cataract Surgery: A Systematic Review and Meta-Analysis

**DOI:** 10.3390/diagnostics12092100

**Published:** 2022-08-30

**Authors:** Matteo Ripa, Nikolaos Kopsacheilis, Kanellina Kanellopoulou, Mikes Nomikarios, Lorenzo Motta

**Affiliations:** 1 Ophthalmology Unit, Fondazione Policlinico Universitario A. Gemelli IRCCS, 00168 Rome, Italy; 2 Ophthalmology Unit, Catholic University “Sacro Cuore”, 00168 Rome, Italy; 3 East Kent Hospitals University NHS Foundation Trust, Kent and Canterbury Hospital Ethelbert Road, Canterbury CT1 3NG, UK; 4 Department of Ophthalmology, William Harvey Hospital, East Kent Hospitals University NHS Foundation Trust, Ashford TN24 0LZ, UK

**Keywords:** Artevo 800, cataract surgery, Ngenuity, 3D Heads-up surgery, TrueVision

## Abstract

Background: The surgical time duration, the postoperative best-corrected visual acuity (BCVA), and the incidence rate of intraoperative complications, alongside the vision and posturing parameters, were estimated by systematic review and meta-analysis to compare the three-dimensional (3D) heads-up visualization system (HUVS) and standard operating microscope (SOM) in cataract surgery. Methods: A literature search was conducted using PubMed, Embase, and Scopus on 26 June 2022. The weighted mean difference (WMD) was used to present postoperative BCVA and the mean surgical time duration, whereas the risk ratio (RR) was used to present the incidence rate of intraoperative complications. Publication bias was evaluated with Egger’s test. The Cochrane Collaboration’s Tool for randomized clinical trials, the methodological index for non-randomized, and the Newcastle-Ottawa Scale were used to assess the risk of bias. The research has been registered with the PROSPERO database (identifier, CRD42022339186). Results: In the meta-analysis of five studies with 1021 participants, the pooled weighted mean difference (WMD) of the postoperative BCVA showed no significant difference between patients who underwent HUVS versus SOM cataract surgery (WMD = −0.01, 95% confidence interval (CI): −0.01 −0.02). In the meta-analysis of nine studies with 5505 participants, the pooled WMD of mean surgical time duration revealed no significant difference between patients who underwent HUVS versus SOM cataract surgery (WMD = 0.17, 95% CI: −0.43–0.76). In the meta-analysis of nine studies with 8609 participants, the pooled risk RR associated with intraoperative complications was 1.00 (95% CI, 1.00–1.01). Conclusions: 3D HUVS and SOM provide comparable surgical time duration, postoperative BCVA, and incidence rate of intraoperative complications.

## 1. Introduction

Over the past two decades, the three-dimensional (3D) heads-up visualization system (HUVS) has been introduced for ophthalmic surgery and is being increasingly adopted by many ophthalmologists [1]. Since the 1990s, many different 3D HUVSs for eye surgeries have been available such as TrueVision^®^ 3D System (TrueVision Systems Inc., Santa Barbara, CA, USA), Sony HD Medical Display System (Sony Electronics, Tokyo, Japan), Ngenuity^®^ 3D Visualization System (Alcon, Fort Worth, TX, USA), and the ARTEVO 800, (Carl Zeiss Meditec AG, Jena, Germany) [2]. Conversely to the surgical operating microscope (SOM), the 3D HUVS allows the surgeon who wears passive polarized 3D glasses to conduct microsurgical operations by looking directly into the 3D panel display rather than at the microscope eyepieces, as the use of passive polarized 3D glasses offers stereopsis [3]. Notably, 3D HUVS uses two high-definition cameras that record image signals from various microscope viewing angles. Soon after, a high-definition 3D screen receives the images that an image processor previously processed [3]. Several studies have widely described the potential advantages of 3D HUVS over traditional SOM. The 3D HUVS offers better ergonomics (the surgeon does not need to bend the neck to look into the microscope eyepieces) and better visualization (high-resolution surgical field visualization with better magnification). Furthermore, 3D imaging is beneficial for teaching, as everyone in the operating room can see the same high-resolution view that the surgeon observes on the screen [4,5,6,7,8,9,10,11,12,13,14]. However, some studies reported different 3D HUVS drawbacks, such as a possible longer surgical time, a challenging learning curve, and a higher surgical workload [9,11]. However, most research focused on vitreoretinal surgery rather than cataract surgery, as the 3D HUVS’s main area of application is still vitreoretinal surgery [15,16]. In addition, no consensus has been reached to measure visual and postural comfort as well as other parameters such as surgeon workload score, maneuverability, teaching potential, and simplicity of use during 3D surgery, thus resulting in many different questionnaires that have been provided to the surgeons to measure these variables. To the best of our knowledge, no published meta-analysis has focused on cataract surgery with 3D HUVS versus standard SOM. Therefore, we conducted a systematic review and meta-analysis of all available studies to investigate further the role of 3D HUVS in cataract surgery and to assess the mean surgical time duration, the postoperative best-corrected visual acuity (BCVA), and the incidence rate of intraoperative complications, alongside the vision and posturing parameters.

## 2. Materials and Methods

### 2.1. Eligibility Criteria

We conducted a systematic review and meta-analysis according to the Preferred Reporting for Systematic Reviews and Meta-Analyses (PRISMA) guidelines [17] and we registered our research with the International Prospective Register of Systematic Reviews (PROSPERO) database (identifier, CRD42022339186). We included comparative studies, such as prospective randomized controlled trials (RCTs), prospective non-RCTs, and retrospective comparative studies that assessed the three-dimensional heads-up visualization systems and the standard operating microscope for cataract surgery.

To be specific, all studies with patients ≥18 years who underwent cataract surgery with either a standard operating microscope or a three-dimensional heads-up visualization system and reporting one of the following outcomes: mean duration of surgical time, the logarithm of the minimum angle of resolution of postoperative BCVA and, the incidence rate of intraoperative complications were included. Articles were excluded if they reported “combined surgery” data such as cataract and glaucoma surgery and combined cataract and vitreoretinal surgery. In addition, literature review studies, conference abstracts, thesis and dissertations; book chapters; technical reports, and letters from the publisher were not included in our analysis. 

Mean surgical time duration in minutes, the postoperative logarithm of the minimum angle of resolution of BCVA, referring to the BCVA within one month after the operation, and incidence rate of intraoperative complications, defined as any complication that occurred during the surgery, such as iris prolapse, posterior capsule rupture, and nucleus drop, were the primary outcomes.

### 2.2. Search Methods

Three databases (PubMed, Embase, and Scopus) were checked from inception until 26 June 2022, using free text to analyze the comparison between the three-dimensional heads-up visualization system and standard operating microscope for cataract surgery. The search strategy combined the keywords according to the indications from each database. The keywords were selected based on readings related to the study’s subject. The keywords were used with Boolean operators to extend and direct the search. For addition and restriction, the Boolean operators OR and AND were used. An asterisk was used to indicate the term was truncated or had a variation in spelling. It was added after the words “head*” and “dimension*” to highlight exposure of interest and widen the search scope. In addition, the investigation was conducted using recognized and extended vocabulary without database filters to achieve a significant sample with a decreased potential loss. Our core search comprised the following terms: “3D” OR “heads-up” AND “cataract” or relevant synonyms, such as “three-dimension”; “cataract extraction”, and “cataract surgery”. To be comprehensive, we accounted for single words and medical terms that could replace the “3D” (“Artevo”; “Ngenuity”, “TrueVision”). This continued until we reached a point when adding more terms provided no new results. In addition, we also hand-searched the bibliographies of included articles to identify further studies that were not found in the initial database search. The detailed search strategy and the PRISMA Checklist are reported in Supplementary Materials S1 and S2.

### 2.3. Study Selection

Two investigators (M.R. and N.K.) independently screened the titles and abstracts of studies to identify articles comparing SOM and 3D HUVS in cataract surgery. In addition, the two reviewers (M.R. and N.K.) also independently reviewed the full text of the remaining studies to assess the study design. Studies with a study design that did not meet the inclusion criteria were excluded.

### 2.4. Data Collection

Two investigators (M.R. and N.K.) independently extracted baseline and outcome data. One co-author (L.M) was consulted for adjudication if consensus could not be reached. Reasons for exclusion were documented. We contacted the corresponding authors of eligible studies whenever the article could not be retrieved, or we needed to obtain additional information that was not available in the article or online Supplementary Files. Thus, the information was extracted directly from the included studies or provided by the corresponding authors. We extracted the following data from each article: the first author, year published, study design, 3D HUVS used, SOM used, number of cataract surgeries, mean surgical time duration, incidence rate of intraoperative complications, postoperative BCVA, mean light exposure time/ocular surface illuminance and other parameters evaluated. We used Covidence systematic review software© (Veritas Health Innovation, Melbourne, Australia), available at www.covidence.org, accessed on 1 July 2022. [18], to record and evaluate the study data until 1 July 2022.

### 2.5. Risk of Bias Assessment

Two authors (M.R. and N.K.) independently appraised the methodological quality of each study by using the Cochrane Collaboration’s Tool for RCT studies [19], and the methodological index for non-randomized studies (MINORS) for non-randomized studies [20]. The risk of bias in observational studies was assessed through the Newcastle-Ottawa Scale (NOS) [21]. Quality assessment data individually appraised by each of the reviewers were compared. If consensus could not be achieved, M.R. and N.K. discussed the discrepancies for adjudication. The data from each reviewer’s quality assessment were compared. M.R. and N.K. discussed the inconsistencies for adjudication if consensus could not be reached.

### 2.6. Assessment of Quality of Evidence

The (Grading of Recommendations Assessment, Development, and Evaluation) GRADE profiler version 3.6 was used to assess the quality of evidence for each outcome, along with the consensus of two authors (M.R. and N.K.) using the GRADE system. The quality of studies is initially rated as high in this system, but it can be downgraded due to (1) bias risk, (2) inconsistency, (3) indirectness, (4) imprecision and (5) publication bias. This system categorizes evidence into four levels of quality: high, moderate, low, and very low [22,23]. Using the GRADE profiler software, the GRADE evidence rating results were recorded in GRADE evidence profiles.

### 2.7. Data Synthesis and Analysis

Our meta-analyses included continuous and dichotomous data from included studies. The risk ratio (RR) was used to present the incidence rate of intraoperative complications, whereas the weighted mean difference (WMD) was used to present postoperative BCVA and the mean surgical time duration. The precision levels of the effect sizes were provided as 95% confidence intervals (CIs). Data were combined using a random-effects model with Mantel Haenszel’s or Inverse variance (IV) method. We assessed and considered between-study heterogeneity as significant if the *p*-value for the Q-test was <0.10 or if the I^2^ statistic was ≥50% [24]. Moreover, the heterogeneity was further investigated by employing Galbraith Plot and sensitivity analysis using the leave-one-out method. Sensitivity analysis was further conducted by excluding studies shown as outliers in the Galbraith plots. If any potential outliers were identified, effect sizes with and without those outliers were reported. Publication bias was assessed using egger’s test. If publication bias was detected, the “trim and fill” method was used to investigate the effect of publication bias. This method conservatively imputes hypothetical negative unpublished studies to mirror the positive studies that cause funnel plot asymmetry. We conducted all analyses using Stata, version 17.0 (StataCorp, College Station, TX, USA). Statistical significance was determined by a two-sided *p*-value of 0.05.

## 3. Results

### 3.1. Study Selection

**Figure 1:**
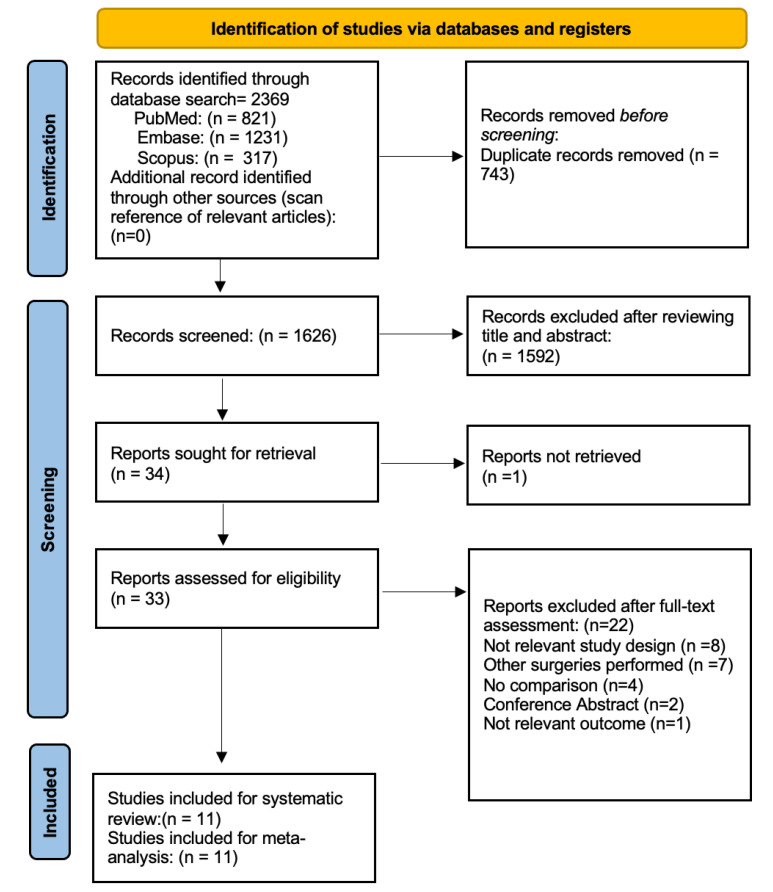
Flow Diagram of the Study Selection Process.

Figure 1 illustrates the flow chart of our analysis’ selection and identification process. The search yielded 2369 indexed articles (821, 1231, and 317 records from PubMed, Embase, and Scopus, respectively). A search of the reference list did not yield other articles. After duplication removal, we screened a total of 1626 articles. After the title and abstract screening, we excluded 1592 studies, and only 34 full-text studies were retrieved and assessed for final eligibility. Indeed, three authors were contacted during the full-text screening stage, but only two responded to requests for additional information to determine eligibility. Furthermore, an additional 22 articles were excluded because the HUVS and SOM were not compared, the comparison involved different kinds of surgeries, or due to the not relevant outcome or not appropriate study design. Finally, a total of 11 studies of 11 articles (8842 eyes) met the inclusion and exclusion criteria and were included in the systematic review and meta-analysis. Notably, nine studies were included in the meta-analysis of postoperative BCVA [4,5,6,8,9,10,11,12,14] and mean surgical time duration [4,5,7,8,9,11,12,13,14], and only five studies were included in the meta-analysis of the incidence rate of intraoperative complications [5,7,9,13,14].

### 3.2. Study Characteristics

A summary of the main characteristics including the first author, year published, study design, 3D HUVS used, SOM used, number of cataract surgeries, mean surgical time duration, the incidence rate of intraoperative complications, postoperative BCVA, mean light exposure time/ocular surface illuminance, and other parameters evaluated are summarized in Table 1. We assessed three RCTs [5,7,13], two non-RCT [9,14], and six comparative observational studies [4,6,8,10,11,12]. The participants ranged from 20 to 3286, with 4816 receiving 3D HUVS cataract surgery and 4026 receiving SOM cataract surgery. Among the studies, three studies [10,11,13] compared the 3D heads-up visualization system and standard operating microscope in different routine ophthalmologic procedures. In contrast, the other eight studies only reported data related to cataract surgery [4,5,6,7,8,9,12,14]. Seven studies used the Ngenuity 3D visualization system [4,6,7,8,11,12,13], whereas the Artevo 800 and the NCVideo 3D system were used in only three studies [5,9,14]. Furthermore, both the Ngenuity 3D visualization system and the Artevo 800 were used in only one study [10]. Regarding the SOM used, three studies reported the Opmi Lumera 700 surgical microscope [6,11,14], and one study the Zeiss Opmi Visu160 surgical microscope [12]. The Oms800 Topcon and the Omi Lumera T surgical microscope were used in only two studies [7,8]. Only two studies assessed “surgical illumination” in terms of ocular surface illuminance and mean light exposure time [6,8]. Several variables were investigated across the studies, such as visual parameters (visual comfort, visibility, depth of field, visibility, detail understanding, image quality), physical discomfort (backache; headache), surgical parameters (operative fluency, maneuverability, simplicity of use, binocular conversion) and cognitive workload [5,7,9,10,11,13,14]. Five studies used a questionnaire to assess these variables at the end of each surgical session [5,10,11,13,14]. Two studies used both phacoemulsification and femtosecond laser cataract surgery [4,12], whereas nine studies reported only phacoemulsification [5,6,7,8,9,10,11,13,14]. In addition, one study reported the assessment of cognitive workload by using real-time tools and self-reports [14].

### 3.3. Postoperative Best-Corrected Visual Acuity

**Figure 2:**
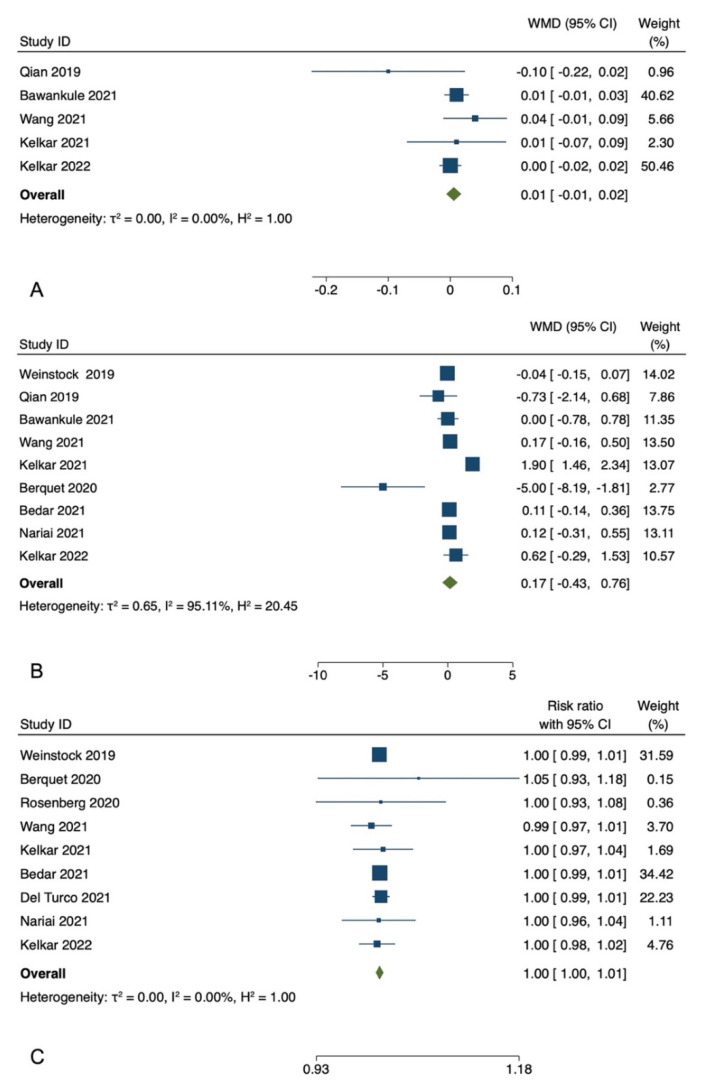
Meta-analyses of the comparison between the three-dimensional heads-up visualization system and the surgical operating microscope for postoperative best-corrected visual acuity (**A**) [5,7,9,13,14], mean surgical duration time (**B**) [4,5,7,8,9,11,12,13,14], and the incidence rate of intraoperative complications (**C**) [4,5,6,8,9,10,11,12,14].

Among the included studies, five studies reported postoperative BCVA [5,7,9,13,14]. The postoperative BCVA showed no significant difference between the treatment and control groups for these studies. In the meta-analysis of five studies with 1021 participants, the pooled WMD of the postoperative BCVA showed no significant difference between patients who underwent HUVS versus SOM cataract surgery (WMD = −0.01, I-V fixed-effects, 95% CI: −0.01 to 0.02, *p* = 0.36; heterogeneity (I^2^) = 0.00%). Egger regression revealed no publication bias with an Egger coefficient of −0.26 (95% CI: −2.75 to 2.22, Prob > |t| = 0.7) (Figure 2).

### 3.4. Duration of Surgical Time

**Figure 3:**
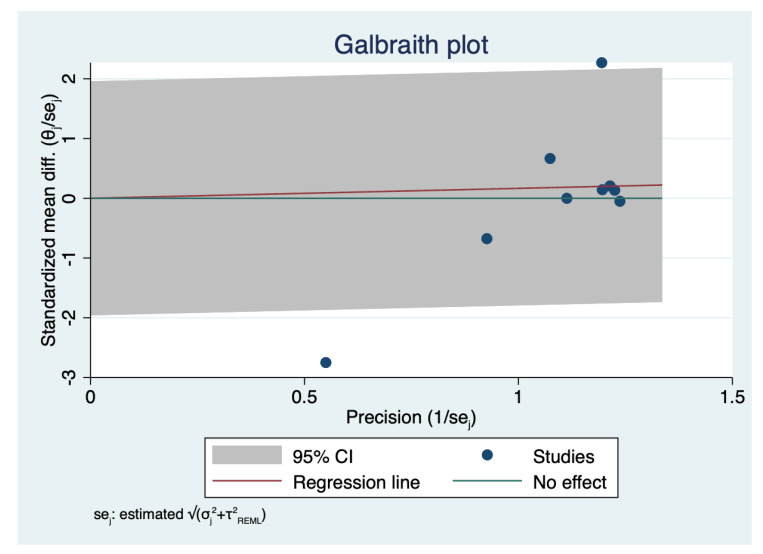
Analysis of heterogeneity across studies: Galbraith plot of mean surgical duration time: The red line depicted the regression line, parallel to the regression line, at a 2-standard-deviation distance, 2 lines (dotted green lines) created an interval in which seven studies (in small circles) fell, indicating 2 potential outliers.

**Figure 4:**
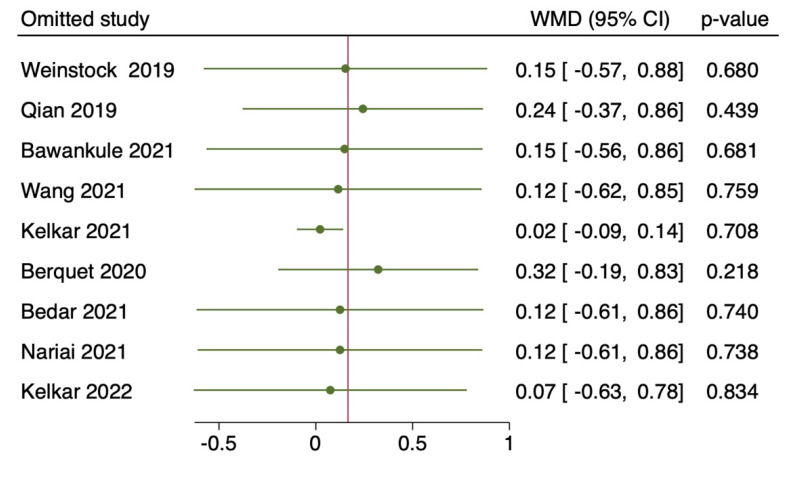
Leave-one-out method: of mean surgical duration time: the horizontal lines and the circles indicate the weighted mean difference and the 95% confidence intervals applying the leave-one-out method [4,5,7,8,9,11,12,13,14].

Nine studies with a total of 5505 eyes, reported the mean surgical duration time [4,5,7,8,9,11,12,13,14]. Specifically, Nariai et al. [8] reported only the operative times for continuous curvilinear capsulorhexis (CCC), phacoemulsification and aspiration (PEA), and irrigation/aspiration (I/A), whereas Bawankule et al. [13] reported both pre-learning and learning phases 3D surgery times. The meta-analysis of nine studies with 5505 participants revealed no statistically significant differences in the pooled WMD of the mean surgical duration time between patients who underwent HUVS versus SOM cataract surgery. (WMD = 0.17, I-V random-effects, 95% CI: −0.43–0.76; I^2^ = 95.11%, *p* = 0.58) (Figure 2). However, there was considerable heterogeneity among the included studies (I^2^ = 95.11%) in this meta-analysis. Thus, we initially conducted a sensitivity analysis to detect outliers and check the influence of individual studies on the stability of the pooled data by excluding one study at a time (“leave-one-out meta-analysis”). The results showed that none of the pooled data were significantly changed by excluding one study at a time. Further examination of study heterogeneity with Galbraith plots used to identify any potential outliers revealed that the mean surgical duration time was influenced by two studies [9,11] (Figure 3 and Figure 4). After outliers’ removal, the heterogeneity was not significant (*p* = 0.61), suggesting the outlier had a large heterogeneity contribution (WMD = 0.03, I-V random-effects, 95% CI: −0.09–0.15; I^2^ = 11.07%, *p* = 0.59). Egger regression revealed publication bias with Egger coefficient of −2.68 (95% CI: −5.15 to −0.19, Prob > |t| = 0.03). After outliers’ removal, Egger regression revealed no publication bias with Egger coefficient of 0.54 (95% CI: −0.93 to 2.02, Prob > |t| = 0.39).

### 3.5. Incidence Rate of Intraoperative Complications

Nine studies with 8609 eyes reported the incidence rate of intraoperative complications, including capsule ruptures, iris prolapse or iris laceration, zonolulolysis, and nucleus drop [4,5,6,8,9,10,11,12,14]. The pooled RR revealed no significant difference in the intraoperative complications between patients who underwent HUVS versus SOM cataract surgery (RR = 1.00, Mantel-Haenszel fixed-effects, 95% CI, 1.00–1.01, *p* = 0.55; I^2^ = 0.0%). Egger regression revealed no publication bias with Egger coefficient of 0.07 (95% CI: −1.21 to 1.34, Prob > |t| = 0.90) (Figure 2).

### 3.6. Risk of Bias and GRADE Assessment

Tables S1–S3 and Table available in Supplementary Material S3 summarise the risk of bias evaluation of all studies. Most of the retrospective comparative studies had a score of 1 or 2 in the major domains of the quality scale used. The quality rating averaged 7.83 ± 0.152 (95% CI 7.71 to 7.96) of the maximum score on the Newcastle-Ottawa Scale. Overall, six studies reached a total score of 8 [4,6,8,11,12], and one reached a total score of 7 [10]. Therefore, there were high study quality and low risk of bias in the included studies. Regarding the RCTs, two studies did not specify the randomization procedure among all included RCTs [7,13]. In contrast, one study established the use of computer-based randomization software that allowed complete concealment of the randomization sequence [5]. In two studies, the method used to conceal the allocation sequence was not described sufficiently [7,13]. One study was registered [5], but the two RCTs were not, leading to reporting bias [7,13]. There were no other potential biases discovered from other sources. The MINORS Scale assessed the risk of bias of the non-RCTs. The studies gained a score of 22 for the lack of a prospective calculation of the study size [9]. The quality of evidence for our primary outcomes (mean surgical duration time, postoperative best-corrected visual acuity, and incidence rate of intraoperative complications) was low according to the GRADE methodology. (Table S1, available in Supplementary Material S3).

## 4. Discussion

To the best of our knowledge, this is the first systematic review and meta-analysis comparing 3D HUVS with SOM in cataract surgery. Analyzing data from 8842 cataract surgeries from eleven studies [4,5,6,7,8,9,10,11,12,13,14], we found that HUVS and SOM are equivalent in terms of the incidence rate of intraoperative complications, mean surgical duration time, and postoperative BCVA.

The duration of the surgery was assessed in nine studies [4,5,7,8,9,11,12,13,14]. Despite similar surgical times, two studies reported significantly different results. Indeed, Berquet et al. [11] showed that the 3D HUVS significantly decreased the duration of cataract surgery, being the first study to detect a significant reduction of a procedure duration using 3D HUVS. However, their limited sample size involved only 25 eyes who underwent 3D HUVS cataract surgery. In contrast, Kelkar et al. [9] reported that the surgical time was significantly higher in the 3D HUVS group. To investigate further, the authors provided a quartile-wise split in surgical times, suggesting that the mean surgical duration time was strictly related to the surgeons’ experience and learning curve, as the surgeons took about one minute less after the first 25 cases. Indeed, the duration decreased from 9.1 ± 1.9 min (first 25 cases) to 8.2 ± 1.9 min (last 25 cases).

The postoperative BCVA and complication rates were similar in the studies included, regardless of the surgical approach [5,7,9,13,14]. This means that 3D HUVS had an equivalent therapeutic effect to SOM in cataract surgery. Therefore, HUVS enables the operation to be safer and more delicate, thereby increasing the procedure’s success rate and avoiding complications [25]. It also improves the visual stereoscopic sense and the function of local image magnification [26]. Kelkar et al. [9] reported that more complications and conversions from 3D HUVS to the SOM occurred in the initial 25 cases, thus highlighting the learning curve’s crucial role.

The 3D HUVS advantages, such as better visual comfort and ergonomics [27,28,29,30], greater operative fluency [2], and educational value [31,32,33], were assessed in the included studies with questionnaires provided to the surgeons at the end of each surgery. However, different scores and assessing modalities were often used for the same variables. Indeed, only two studies used a previously published questionnaire that analyzed comfort, visibility, image quality, depth perception, simplicity of use, maneuverability, and teaching using a scale of 1 to 5: (1 = low; 2 = below average; 3 = average; 4 = good; and 5 = excellent) [5,10]. Instead, different categorical evaluations, scales, or open-ended fashions were used in the other included studies [9,11,13]. Nonetheless, the 3D HUVS had a significantly higher rating of satisfaction than the SOM for each parameter in all studies except one where no statistical difference for visual comfort, operative fluency, backache, and headache was found [11]. Indeed, the field of view is better in 3D HUVS as it offers 30–40% higher magnification than SOM [26]; the depth of field offered by HUVS is higher even under high magnification regardless of the area focused [32,34,35,36]; the 3D HUVS ergonomic design provides a more physiologically comfortable and stable body posture relieving fatigue and musculoskeletal stress [2,37,38,39]. Furthermore, Kelkar et al. [14] assessed the cognitive workload using a perioperative real-time tool and the surgery task load index (SURG-TLX) questionnaire at the end of the surgery. They found no differences between 3D HUVS and SOM in the surgeons’ heart rate, oxygen saturation levels, surgery task load index total workload score, and workload score for all six dimensions of the questionnaire. Moreover, Rosenberg et al. [6] demonstrated that the light intensity was significantly decreased in patients who underwent 3D HUVS cataract surgery compared to those who underwent SOM surgery and Nariai et al. [8] soon after stated 3D HUVS cataract surgery required lower light intensity than cataract surgery with a standard microscope eyepiece. Therefore, cataract surgery with a 3D HUVS may reduce the risk of retinal phototoxicity and photophobia.

Although Kelkar et al. [9] reported a higher performance after the first twenty-five 3D HUVS surgeries, Berquet et al. [11] emphasized that all surgeons found the learning curve to be fast, reaching good comfort and efficiency just after three or four surgeries. However, many surgeons felt less comfortable in cataract surgeries than in posterior segment surgeries [11]. This may be due to the image latency period that had a more pronounced impact in the anterior procedures due to the higher instrumental speed during surgical manipulations. Indeed, the human brain cannot recognize a time lag (the period of time between performing an action and its visibility on the screen) </=50 ms [26]. To further investigate the time lag effect, Wang et al. [5] compared this variable between SOM and 3D HVUS groups during each cataract surgery step, demonstrating that the time lag was appreciated only during the learning phase, but soon resolved during the post-learning phase. 

Our systematic review and meta-analysis’ strength is the high volume of cataract surgeries assessed. Nevertheless, this systematic review and meta-analysis have several limitations. First, we only analyzed a maximum of eight studies in each meta-analysis. Second, we could not run a meta-analysis of physical discomfort, and surgical and visual parameters as these variables cannot be easily analyzed. Third, we found high heterogeneity in the mean surgical duration time, which was explained by outliers’ removal. Fourth, we did not analyze the cost of the 3D HUVS systems. Fifth, few papers deeply analyzed the learning curve parameters. Sixth, we included studies with a different design. Therefore, the estimated intervention effects might be influenced to varying degrees by different sources of bias. Finally, we also recruited studies that did not analyze “combination surgery” parameters.

## 5. Conclusions

Our meta-analyses synthesized the currently available evidence regarding the comparison of 3D HUVS and SOM for cataract surgery. The recruited studies included in our systematic review further suggest that 3D HUVS provides comparable mean surgical duration time, postoperative BCVA, and incidence of complication rate to SOM, despite several advantages in terms of visual and postural comforts.

## Figures and Tables

**Table 1:** Characteristics of studies included in the systematic review and meta-analyses.

Study	Year	Study Design	3D HUVS Used	SOM Used	Number of Cataract Surgeries (n.)	Surgical Duration: (min.)	Intraoperative Complications:(n.)	Postoperative BCVA (LogMar)	Mean Light Exposure Time (min.)/Ocular Surface Illuminance (lux)	Other Parameters Evaluated
Weinstock et al.	2019	RS	Ngenuity 3D visualization system	Not reported	Total: 2320 HUVS: 1673 SOM: 647	HUVS: 6.48 ± 1.15 SOM: 6.52 ± 1.38	Total: 17 HUVS: 12 SOM: 5	Not reported	Not reported	None
Qian et al.	2019	RCT	Ngenuity 3D visualization system	OPMI LUMERA T surgical microscope	Total: 20 HUVS: 10 SOM: 10	HUVS: 8.3 ± 1.73 SOM:9.03 ± 1.47	Not reported	HUVS: 0.19 ± 0.2 SOM: 0.09 ± 0.14	Not reported	Preoperative BCVA and Preoperative and Postoperative ECD
Berquet et al.	2020	RS	Ngenuity 3D visualization system	OPMI LUMERA 700	Total: 73 HUVS: 25 SOM: 48	HUVS: 16.44 ± 4.36 SOM: 21.44 ± 7.50	Total: 5 HUVS: 1 SOM: 4	Not reported	Not reported	Visual comfort; Operative Fluency; Backache; Headache
Rosenberg et al.	2020	RS	Ngenuity 3D visualization system	OPMI LUMERA 700	Total: 51 HUVS: 27 SOM: 24	Not reported	Total: 0 HUVS: 0 SOM: 0	UDVA in lines of vision gained	HUVS: 23.8 ± 1.9 SOM: 25.8 ± 3.0	None
Bawankule et al.	2021	RCT	Ngenuity 3D visualization system	Not reported	Total: 213 HUVS: 101 SOM: 112	HUVS: 13.11 ± 3.7 (learning phase) 9.74 ± 2.79 (post-learning phase) SOM: 9.74 ± 2.97	Not reported	HUVS: 0.02 ± 0.06 (learning Phase) 3D: 0.03 ± 0.07 (Post-learning Phase) SOM: 0.03 ± 0.07	Not reported	Surgical outcomes surgeon’s perspective like time lag, illumination, learning curve, ease of doing various steps and its value as an educational tool
Wang et al.	2021	RCT	NCVideo 3D system	Not reported	Total: 242 HUVS: 117 SOM: 125	HUVS: 7.7 ± 1.34 SOM:7.53 ± 1.28	Total: 1 HUVS: 1 SOM: 0	HUVS: 0.26 ± 0.2 SOM: 0.30 ± 0.2	Not reported	Depth of field, visibility, detail understanding; knowledge retention; and educational value; preoperative BCVA and preoperative and postoperative IOP
Kelkar et al.	2021	Non-RCT	Artevo 800	Not reported	Total: 343 HUVS: 100 SOM: 243	HUVS: 8.4 ± 2.1 SOM: 6.5 ± 1.8	Total: 8 HUVS: 2 SOM: 6	SOM 0.40 ± 0.27 HUVS: 0.41 ± 0.34	Not reported	Surgical parameters; binocular conversion, Difficulty with 3-D Heads up display group (low illumination, difficult depth perception) and surgeon workload score
Bedar et al.	2021	RS	Ngenuity 3D visualization system	Zeiss OPMI Visu160	Total: 2000 HUVS: 1000 SOM: 1000	HUVS: 11.84 ± 2.9 SOM: 11.73 ± 2.8	Total: 15 HUVS: 7 SOM: 8	Not reported	Not reported	None
Del Turco et al.	2021	RS	Ngenuity 3DVisualization System and Artevo 800	Not reported	Total: 3286 HUVS: 1638 SOM: 1648	Not reported	Total: 62 HUVS: 29 SOM: 33	Not reported	Not reported	Comfort, visibility, image quality, maneuverability, teaching potential, depth perception, and simplicity of use
Nariai et al.	2021	RS	Ngenuity 3D visualization system	OMS800 Topcon	Total: 91 HUVS: 45 SOM: 46	HUVS: CCC + PEA + I/A: 3.2 ± 1.16 SOM: CCC + PEA + I/A: 3.08 ± 0.94	Total: 0 HUVS: 0 SOM: 0	Not reported	Ocular surface illuminance HUVS: 5500 ± 2000 SOM: 1900 ± 1800	None
Kelkar et al.	2022	Non-RCT	Artevo 800	Zeiss Lumera 700	Total: 203 HUVS: 80 SOM: 123	HUVS: 8.07 ± 2.94 SOM: 7.45 ± 3.66	Total: 0 HUVS: 0 SOM: 0	SOM: 0.3 (0.2–0.48) HUVS: 0.3 (0.2–0.5)	Not reported	Cognitive load assessment (Heart Rate, Oxigen Saturation and SURG-TLX analysis)

Abbreviations: N: number; Min: minute; IOP: Intraocular pressure, HUVS: Head-up visualization system; SOM: surgical operating microscope; RS: retrospective comparative study; PS: prospective comparative study; non-RCT: non randomized clinical trial; RCT: randomized clinical trial; 3D: three dimensional; BCVA: best-correct visual acuity; UDVA: uncorrect distance visual acuity; ECD: endothelial cell density; CCC: central curvilinear capsulorhexis; PEA: phacoemulsification and aspiration; I/A: irrigation and aspiration; SURG-TLX: surgery task load index.

## Data Availability

Not applicable.

## Supplementary Materials

The following supporting information can be downloaded at: https://www.mdpi.com/article/10.3390/diagnostics12092100/s1, Supplementary Material S1: Detailed Search Strategy. Supplementary Material S2: PRISMA Checklist. Supplementary Material S3: Risk of bias and GRADE assessment.

## References

[1] Moura-Coelho N., Henriques J., Nascimento J., Dutra-Medeiros M. (2019). Three-Dimensional Display Systems in Ophthalmic Surgery—A Review. Eur. Ophthalmic Rev..

[2] Savastano A., Ripa M., Savastano M.C., de Vico U., Caporossi T., Kilian R., Rizzo S. (2021). Comparison of Novel Digital Microscope Using Integrated Intraoperative OCT with Ngenuity 3D Visualization System in Phacoemulsification. Can. J. Ophthalmol..

[3] Liu J., Wu D., Ren X., Li X. (2021). Clinical Experience of Using the NGENUITY Three-Dimensional Surgery System in Ophthalmic Surgical Procedures. Acta Ophthalmol..

[4] Weinstock R.J., Diakonis V.F., Schwartz A.J., Weinstock A.J. (2019). Heads-up Cataract Surgery: Complication Rates, Surgical Duration, and Comparison with Traditional Microscopes. J. Refract. Surg..

[5] Wang K., Song F., Zhang L., Xu J., Zhong Y., Lu B., Yao K. (2021). Three-Dimensional Heads-up Cataract Surgery Using Femtosecond Laser: Efficiency, Efficacy, Safety, and Medical Education-A Randomized Clinical Trial. Transl. Vis. Sci. Technol..

[6] Rosenberg E.D., Nuzbrokh Y., Sippel K.C. (2021). Efficacy of 3D Digital Visualization in Minimizing Coaxial Illumination and Phototoxic Potential in Cataract Surgery: Pilot Study. J. Cataract Refract. Surg..

[7] Qian Z., Wang H., Fan H., Lin D., Li W. (2019). Three-Dimensional Digital Visualization of Phacoemulsification and Intraocular Lens Implantation. Indian J. Ophthalmol..

[8] Nariai Y., Horiguchi M., Mizuguchi T., Sakurai R., Tanikawa A. (2021). Comparison of Microscopic Illumination between a Three-Dimensional Heads-up System and Eyepiece in Cataract Surgery. Eur. J. Ophthalmol..

[9] Kelkar J.A., Kelkar A.S., Bolisetty M. (2021). Initial Experience with Three-Dimensional Heads-up Display System for Cataract Surgery—A Comparative Study. Indian J. Ophthalmol..

[10] del Turco C., D’Amico Ricci G., Dal Vecchio M., Bogetto C., Panico E., Giobbio D.C., Romano M.R., Panico C., la Spina C. (2022). Heads-up 3D Eye Surgery: Safety Outcomes and Technological Review after 2 Years of Day-to-Day Use. Eur. J. Ophthalmol..

[11] Berquet F., Henry A., Barbe C., Cheny T., Afriat M., Benyelles A.K., Bartolomeu D., Arndt C. (2020). Comparing Heads-Up versus Binocular Microscope Visualization Systems in Anterior and Posterior Segment Surgeries: A Retrospective Study. Int. J. Ophthalmol..

[12] Bedar M.S., Kellner U. (2022). Digital 3D “Heads-up” Cataract Surgery: Safety Profile and Comparison with the Conventional Microscope System. Klin. Mon. Fur Augenheilkd..

[13] Bawankule P., Narnaware S., Chakraborty M., Raje D., Phusate R., Gupta R., Rewatkar K., Chivane A., Sontakke S. (2021). Digitally Assisted Three-Dimensional Surgery—Beyond Vitreous. Indian J. Ophthalmol..

[14] Kelkar A., Kelkar J., Chougule Y., Bolisetty M., Singhvi P. (2022). Cognitive Workload, Complications and Visual Outcomes of Phacoemulsification Cataract Surgery: Three-Dimensional versus Conventional Microscope. Eur. J. Ophthalmol..

[15] Agranat J.S., Douglas V.P., Douglas K.A.A., Miller J.B. (2020). A Guarded Light Pipe for Direct Visualization during Primary Scleral Buckling on the Ngenuity Platform. Int. J. Retin. Vitr..

[16] Romano M.R., Cennamo G., Comune C., Cennamo M., Ferrara M., Rombetto L., Cennamo G. (2018). Evaluation of 3D Heads-up Vitrectomy: Outcomes of Psychometric Skills Testing and Surgeon Satisfaction. Eye.

[17] Moher D., Liberati A., Tetzlaff J., Altman D.G. (2009). Preferred Reporting Items for Systematic Reviews and Meta-Analyses: The PRISMA Statement. BMJ.

[18] Cleo G., Scott A.M., Islam F., Julien B., Beller E. (2019). Usability and Acceptability of Four Systematic Review Automation Software Packages: A Mixed Method Design. Syst. Rev..

[19] Higgins J.P.T., Altman D.G., Gøtzsche P.C., Jüni P., Moher D., Oxman A.D., Savović J., Schulz K.F., Weeks L., Sterne J.A.C. (2011). The Cochrane Collaboration’s Tool for Assessing Risk of Bias in Randomised Trials. BMJ.

[20] Slim K., Nini E., Forestier D., Kwiatkowski F., Panis Y., Chipponi J. (2003). Methodological Index for Non-Randomized Studies (Minors): Development and Validation of a New Instrument. ANZ J. Surg..

[21] Lo C.K.L., Mertz D., Loeb M. (2014). Newcastle-Ottawa Scale: Comparing Reviewers’ to Authors’ Assessments. BMC Med. Res. Methodol..

[22] Balshem H., Helfand M., Schünemann H.J., Oxman A.D., Kunz R., Brozek J., Vist G.E., Falck-Ytter Y., Meerpohl J., Norris S. (2011). GRADE Guidelines: 3. Rating the Quality of Evidence. J. Clin. Epidemiol..

[23] Guyatt G.H., Oxman A.D., Vist G.E., Kunz R., Falck-Ytter Y., Alonso-Coello P., Schünemann H.J. (2008). GRADE: An Emerging Consensus on Rating Quality of Evidence and Strength of Recommendations. BMJ.

[24] Higgins J.P.T., Thompson S.G. (2002). Quantifying Heterogeneity in a Meta-Analysis. Stat. Med..

[25] Rani D., Kumar A., Chandra P., Chawla R., Hasan N., Agarwal D., Prasad R. (2021). Heads-up 3D Viewing System in Rhegmatogenous Retinal Detachment with Proliferative Vitreoretinopathy—A Prospective Randomized Trial. Indian J. Ophthalmol..

[26] Eckardt C., Paulo E.B. (2016). Heads-Up Surgery for Vitreoretinal Procedures. Retina.

[27] Kumar A., Hasan N., Kakkar P., Mutha V., Karthikeya R., Sundar D., Ravani R. (2018). Comparison of Clinical Outcomes between “Heads-up” 3D Viewing System and Conventional Microscope in Macular Hole Surgeries: A Pilot Study. Indian J. Ophthalmol..

[28] Coppola M., la Spina C., Rabiolo A., Querques G., Bandello F. (2017). Heads-up 3D Vision System for Retinal Detachment Surgery. Int. J. Retin. Vitr..

[29] Zhang Z., Wang L., Wei Y., Fang D., Fan S., Zhang S. (2019). The Preliminary Experiences with Three-Dimensional Heads-Up Display Viewing System for Vitreoretinal Surgery under Various Status. Curr. Eye Res..

[30] Palácios R.M., de Carvalho A.C.M., Maia M., Caiado R.R., Camilo D.A.G., Farah M.E. (2019). An Experimental and Clinical Study on the Initial Experiences of Brazilian Vitreoretinal Surgeons with Heads-up Surgery. Graefes Arch. Clin. Exp. Ophthalmol..

[31] Rizzo S., Abbruzzese G., Savastano A., Caporossi T., Barca F. (2018). 3D Surgical viewing system in ophthalmology: Perceptions of the Surgical Team. Retina.

[32] Mendez B.M., Chiodo M.V., Vandevender D., Patel P.A. (2016). Heads-up 3D Microscopy: An Ergonomic and Educational Approach to Microsurgery. Plast. Reconstr. Surg. Glob. Open.

[33] De J., Barbosa P., Ribeiro J.C., Machado A.J. (2021). Heads-up Surgery with Three-Dimensional Display Devices for Cataract Surgeries Cirurgia Heads-up Com Sistemas de Visualização Tridimensional Para Cirurgias Oftálmicas de Catarata. Arq. Bras. Oftalmol..

[34] Ohno H. (2019). Utility Of Three-Dimensional Heads-Up Surgery In Cataract And Minimally Invasive Glaucoma Surgeries. Clin. Ophthalmol..

[35] Hamasaki I., Shibata K., Shimizu T., Kono R., Morizane Y., Shiraga F. (2019). Lights-out Surgery for Strabismus Using a Heads-Up 3D Vision System. Acta Med. Okayama.

[36] Galvis V., Berrospi R.D., Arias J.D., Tello A., Bernal J.C. (2017). Heads up Descemet Membrane Endothelial Keratoplasty Performed Using a 3D Visualization System. J. Surg. Case Rep..

[37] Reddy S., Mallikarjun K., Mohamed A., Mruthyunjaya P., Dave V.P., Pappuru R.R., Chhablani J., Narayanan R. (2021). Comparing Clinical Outcomes of Macular Hole Surgeries Performed by Trainee Surgeons Using a 3D Heads-up Display Viewing System versus a Standard Operating Microscope. Int. Ophthalmol..

[38] Gupta Y., Tandon R. (2022). Optimization of Surgeon Ergonomics with Three-Dimensional Heads-up Display for Ophthalmic Surgeries. Indian J. Ophthalmol..

[39] Kaur M., Titiyal J.S. (2020). Three-Dimensional Heads up Display in Anterior Segment Surgeries- Expanding Frontiers in the COVID-19 Era. Indian J. Ophthalmol..

