# Supplementary material for: Three-Dimensional Heads-Up vs. Standard Operating Microscope for Cataract Surgery: A Systematic Review and Meta-Analysis
Source: Diagnostics (Basel). 2022 Aug 30;12(9):2100. doi: 10.3390/diagnostics12092100 (PMC9497825; doi:10.3390/diagnostics12092100)
Supplement: Supplementary file 1 [file diagnostics-12-02100-s001.zip › Supplementary Material S1.pdf]

Supplementary Material S1: Detailed Search Strategy

Free Text
Initial search performed on 16 June 2022
Search updated on 26 June 2022
No limits applied

#	Search Term	No. Results
1	3D OR (three AND dimension*) OR (3-dimension*) OR (three-dimension*)	510,770
2	Head*-up OR (head* AND up)	100,307
3	“Artevo” OR “Ngenuity” OR “TrueVision”	50
4	1 OR 2 OR 3	608,253
7	“Cataract OR (Cataract AND surgery) OR (Cataract AND extraction)	84,972
7	6 AND 7	821

Embase

Free Text
Initial search performed on 16 June 2022
Search updated on 26 June 2022
[embase]/lim, no other limits applied

Mapping options enabled:

- map to preferred term in Emtree
- search also as free text in all fields
- explode using narrower Emtree terms

search as broadly as possible

#	Search Term	No. of Results
1	3D OR (three AND dimension*) OR (3-dimension*) OR (three-dimension*) OR heads-up OR (head AND up) OR “Artevo” OR “Ngenuity” OR “TrueVision AND “Cataract OR (Cataract AND surgery) OR (Cataract AND extraction)”	1231

Scopus
Free Text
Initial search performed on 16 June 2022
Search updated on 26 June 2022

#	Search Term	No. of Results
1	(TITLE-ABS-KEY (3d OR (three AND dimension*) OR (3-dimension*) OR (three- AND dimension*)) OR TITLE-ABS-KEY (heads-up OR (head AND up)) OR TITLE-ABS-KEY ("Artevo" OR "Ngenuity" OR "TrueVision")) AND (TITLE-ABS KEY (cataract OR cataract AND surgery OR cataract AND extraction))	317
