# Supplementary material for: Three-Dimensional Heads-Up vs. Standard Operating Microscope for Cataract Surgery: A Systematic Review and Meta-Analysis
Source: Diagnostics (Basel). 2022 Aug 30;12(9):2100. doi: 10.3390/diagnostics12092100 (PMC9497825; doi:10.3390/diagnostics12092100)
Supplement: Supplementary file 1 [file diagnostics-12-02100-s001.zip › Supplementary Material S3.pdf]

Table S1: Newcastle–Ottawa Scale for Critical Appraisal of Case-Control Studies

	Selection				Comparability	Exposure			Total
Source	Adequate case definition	Representativeness of the cases	Selection controls	Definitios of controls	Comparability of cases and controls	Ascertainment of exposure	Same method of ascertainment for cases and controls	Non-Response rate	
Weinstock et al., 2019	+1 (a)	+1 (a)	+1 (a)	+1 (a)	+1 (a)	+1 (a)	+1 (a)	+1 (a)	8/10
Berquet et al., 2020	+1 (a)	+1 (a)	+1 (a)	+1 (a)	+1 (a)	+1 (a)	+1 (a)	+1 (a)	8/10
Rosenberg et al., 2020	+1 (a)	+1 (a)	+1 (a)	+1 (a)	+1 (a)	+1 (a)	+1 (a)	+1 (a)	8/10
Bedar et al., 2021	+1 (a)	+1 (a)	+1 (a)	+1 (a)	+1 (a)	+1 (a)	+1 (a)	+1 (a)	8/10
Del Turco et al., 2021	+1 (a)	+1 (a)	+1 (a)	+1 (a)	+1 (a)	0 (c)	+1 (a)	+1 (a)	7/10
Nariai et al., 2022	+1 (a)	+1 (a)	+1 (a)	+1 (a)	+1 (a)	+1 (a)	+1 (a)	+1 (a)	8/10

Selection: 1) Is the case definition adequate? a) yes, with independent validation, b) yes, eg record linkage or based on self reports, c) no description 2) Representativeness of the cases: a) consecutive or obviously representative series of cases, b) potential for selection biases or not stated3) Selection of Controls: a) community controls, b) hospital controls, c) no description, 4) Definition of Controls a) no history of disease (endpoint); b) no description of source; Comparability: 1) Comparability of cases and controls on the basis of the design or analysis: a) study controls for (Select the most important factor.), b) study controls for any additional factor; Exposure: 1) Ascertainment of exposure a) secure record (eg surgical records), b) structured interview where blind to case/control status, c) interview not blinded to case/control status, d) written self report or medical record only e) no description; 2) Same method of ascertainment for cases and controls; a) yes, b) no; 3) Non-Response rate: a) same rate for both groups; b) non respondents described; c) rate different and no designation.

Table S2: Risk of bias for randomized controlled trial studies using Cochrane Collaboration's Tool

	Selection Bias		Performance Bias	Detection Bias	Attrition Bias	Reporting Bias	Other
Source	Random sequence generation	Allocation concealment	Blinding of participants and personnel	Blinding of outcome assessment	Incomplete outcome data addressed	Selective reporting	Other bias
Qian et al., 2019	Unclear	Unclear	High	High	Unclear	Low	Low
Bawankule et al., 2020	Unclear	Unclear	High	High	Unclear	Low	Low
Wang et al., 2021	Low	Low	High	High	Unclear	Low	Low

Table S3: Risk of bias non- randomized controlled trial studies using Methodological Index for Non-randomized Studies (MINORS) Scale

Study	A clearly stated aim	Inclusion of consecutive patients	Prospective collection of data	Endpoints appropriate to the aim of the study	Unbiased assessment of the study endpoint	Follow-up period appropriate to the aim of the study	Loss to follow up less than 5%	Prospective calculation of the study size	An adequate control group	Contemporary groups	Baseline equivalence of groups	Adequate statistical analyses	Total
Kelkar et al., 2021	2	2	2	2	2	2	2	0	2	2	2	2	22
Kelkar et al., 2022	2	2	2	2	2	2	2	0	2	2	2	2	22

The items are scored 0 (not reported), 1 (reported but inadequate) or 2 (reported and adequate).

Table S4: GRADE assessment

Certainty assessment								Patients (n)		Effect	Certainty
**Outcome**	**Studies (n)**	**Study design**	**Risk of bias**	**Inconsistency[a]**	**Indirectness[d]**	**Imprecision[c]**	**Publication bias[b]**	**Group 1**	**Group 2**	**Relative MD or OR (95% CI)**	
Duration of surgical time	9	observational studies RCT and Non-RCT	not serious	serious	not serious	Not serious	Undetected	3151	2354	**0.17** (-0.43 to 0.76)	⊕⊕◯◯ Low
Postoperative best-corrected visual acuity	5	observational studies RCT and Non-RCT	not serious	Not serious	not serious	Not serious	Undetected	408	613	**0.01** (-0.01 to 0.02)	⊕⊕◯◯ Low
Intraoperative complications	9	observational studies RCT and Non-RCT	not serious	Not serious	not serious	Not serious	Undetected	4705	3904	**1.00** (1.00 to 1.01)	⊕⊕◯◯ Low

CI: Confidence interval; **MD:** Mean difference; **OR:** Odds ratio; [a] Substantial heterogeneity I^2 > 60% (serious) or >90% (very serious); [b] Strongly suspected if funnel plot suggestive of publication bias or lack of small studies and negative effects; [c] serious if total number of events is less than 300, CIs overlap or non clinically significant effect; [d] Serious indirectness refer to variation of outcome measure or definition across studies
